# Multimodal MRI markers of white matter hyperintensities and their impact on cognition in cerebral small vessel disease: a systematic review

**DOI:** 10.1007/s11682-026-01209-8

**Published:** 2026-10-08

**Authors:** Babina Aryal, Saikiran Pendem, Priya P.S., Rajagopal Kadavigere, Priyanka, Rahul Ranjan

**Affiliations:** 1 Department of Medical Imaging Technology, Manipal College of Health Professions, Manipal Academy of Higher Education, Manipal, India; 2 Department of Radiodiagnosis and Imaging, Kasturba Medical College, Manipal Academy of Higher Education, Manipal, India; 3 Department of Radio-diagnosis, Rama Medical College, Kanpur, India

**Keywords:** Cerebral small vessel disease, White matter hyperintensities, Magnetic resonance imaging, Cognitive impairment, Neuroimaging biomarkers

## Abstract

Cerebral small vessel disease (CSVD) is a leading cause of stroke, dementia, and age-related cognitive decline, with white matter hyperintensities (WMH) being a key imaging feature in MRI. This review aims to evaluate the association between multimodal magnetic resonance imaging markers of white matter hyperintensities and cognition in cerebral small vessel disease. A systematic search of PubMed, Scopus, Embase, and Web of Science (2016–2026) was done. Studies involving adults with MRI visible CSVD markers who underwent MRI standardized cognitive assessment were eligible. Fifteen studies were included with a total of 5752 participants. Included studies used conventional and advanced MRI techniques to evaluate the association of WMH with cognitive function. The findings were synthesized narratively according to MRI techniques. Conventional MRI studies showed that greater WMH burden and CSVD severity were associated with poorer cognition, particularly processing speed and global cognition, with individual studies reporting β values of − 0.371 (*p* < 0.001) and − 0.363 (*p* < 0.001), with good predictive accuracy reported in one study (AUC = 0.800, 95% CI: 0.710–0.891). While Advanced MRI also showed associations with microstructural and microvascular damage in white matter and with reduced blood flow was associated with poorer cognitive performance, individual studies reported β values of 0.268–0.273, moderate-to-strong associations (*r* = 0.310–0.441), good diagnostic accuracy (AUC = 0.86–0.89), and significantly lower scores on cognitive assessments (Mini-Mental State Examination: *p* < 0.001; Montreal Cognitive Assessment: *p* < 0.05). These findings suggest that WMH burden and disrupted white matter microstructure are associated with cognitive impairment. Larger, standardized studies are needed to determine the clinical value of MRI markers as reliable biomarkers for early detection and monitoring of cognitive impairment in CSVD.

**Supplementary Information:**

The online version contains supplementary material available at https://doi.org/10.1007/s11682-026-01209-8.

## Introduction

Cerebral Small Vessel Disease (CSVD) is a leading cause of 25% of strokes and nearly 45% of dementia and an important cause of age-related cognitive decline (Pantoni, 2010). It involves a pathological change in small cerebral vessels, typically measuring approximately 50–400 μm in diameter, including alterations in vessel structure and function that can impair the blood- brain barrier and cerebral perfusion (Chojdak-Łukasiewicz et al., 2021). The global prevalence of CSVD is rising; therefore, early detection of individuals at risk of cognitive impairment is essential. Magnetic Resonance Imaging (MRI) is the preferred modality for evaluating CSVD, as it is sensitive to the detection of the structure and function of the brain. Studies have shown that CSVD is associated with parenchymal changes that appear as white matter hyperintensities (WMHs), lacunar infarcts, cerebral microbleeds, and increased perivascular spaces within the basal ganglia region, hippocampus, and brain atrophy, with the prevalence of WMH increasing with age from 5% at 50 years to near 100% at 90 years (Gertje et al., 2021; Mahammedi et al., 2022; Wardlaw et al., 2013). Conventional MRI sequence fluid-attenuated inversion recovery (FLAIR) detects the structural abnormalities seen in CSVD lesions frequently observed in older adults but is less sensitive in detecting early microstructural damage that occurs before visible lesions (Wardlaw et al., 2019).

Multiple studies have shown that WMH are associated with cognitive decline, which affects executive function, attention, memory, and processing speed (Arvanitakis et al., 2016; Qi et al., 2019). Studies have also demonstrated that microstructural and physiological alterations extend beyond visible lesions into the surrounding normal-appearing white matter (NAWM), often referred to as the WMH “penumbra” (Wu et al., 2019). Recent advances in neuroimaging techniques have improved the ability to detect subtle microstructural and microvascular abnormalities, even when they appear normal on conventional MRI. Prior cross-sectional neuroimaging studies have shown that structural lesions, such as WMH and lacunes detected on advanced MRI, are strongly associated with cognitive impairment. Diffusion-based imaging, including Diffusion Tensor Imaging and Diffusion Kurtosis Imaging, sensitively detects white matter microstructural damage and subtle alterations in patients with CSVD (Hu et al., 2022; Pasi et al., 2016). Additionally, advanced perfusion and diffusion MRI techniques, such as arterial spin labeling (ASL) and intravoxel incoherent motion (IVIM), have also shown reduced blood flow and microvascular and microstructural changes in WMH and normal-appearing white matter (NAWM) (Neumann et al., 2022; Wang et al., 2025; Wong et al., 2017).

Although previous studies have investigated the association between individual MRI markers and cognition in CSVD, a comprehensive synthesis of evidence linking WMH and cognitive outcomes across different MRI techniques remains limited. This systematic review therefore focused on the association between WMH and cognitive performance in CSVD using conventional and advanced MRI techniques, including structural MRI, DTI, ASL, IVIM, and DKI.

## Materials and methods

This protocol was prepared as per the Preferred Reporting Items for Systematic Reviews and Meta-Analyses (PRISMA) guidelines and registered in PROSPERO (CRD420261476616).

### Information sources and search strategy

A literature search was conducted in PubMed, Scopus, Embase, and Web of Science to identify relevant studies. The search was limited to studies published between 2016 and 2026 to focus on recent evidence and developments in the MRI based assessment of CSVD and its relationship with cognitive function. Search terms used were “cerebral small vessel disease,” “white matter hyperintensities,” “magnetic resonance imaging,”, “FLAIR,” “arterial spin labeling,” “diffusion tensor imaging,” “diffusion kurtosis imaging,” “intravoxel incoherent motion,” and “cognitive impairment”. Boolean operators “AND” and “OR” were used. A detailed description of the search strategy including search terms, Boolean Operators and applied limits is provided in Supplementary Material 1.

### Inclusion criteria and study selection process

The inclusion criteria for this review were prepared according to PECO (Population, Exposure, Comparator, and Outcome) framework. The detailed screening process for determining study criteria is shown in Table 1. This review included original peer-reviewed studies that investigated the association between neuroimaging markers of cerebral small vessel disease (CSVD), particularly white matter hyperintensities (WMH), and cognitive function. Studies involving older adult participants with MRI visible evidence of CSVD underwent standardized cognitive assessment were considered eligible, irrespective of whether they had a clinical diagnosis of CSVD, including those from community based or cognitively normal populations. Both conventional and advanced MRI techniques were included, provided that the studies examined the relationship between imaging findings and cognitive outcomes. Studies including participants with lacunar stroke or transient ischemic attack (TIA) were retained when the primary focus was on CSVD imaging markers and cognitive function, consistent with the predefined eligibility criteria. Only peer-reviewed original research articles that reported relevant quantitative imaging data were included. Studies involving neurological disorders other than CSVD, such as brain tumours, Alzheimer’s disease, Parkinson’s disease and multiple sclerosis as well as reviews, editorials, conference papers, and case reports, were excluded. Rayyan software was used for the literature search and elimination of duplicates. Screening based on titles, abstracts, and full texts was conducted independently by two reviewers, with disagreements resolved by a third reviewer.

**Table 1:** PECO criteria for study selection

PECO Component	Criteria
Population	Adults with MRI CSVD, including WMH, from clinical or community-based populations.
Exposure	MRI-visible CSVD markers, particularly WMH, assessed using conventional and advanced MRI techniques, including T1, T2, FLAIR, DTI, DKI, IVIM, ASL, and DWI.
Comparator	Individuals with lower or absent CSVD/WMH burden, or healthy controls, where applicable.
Outcome	Cognitive function or cognitive impairment assessed using standardized cognitive measures, and its association with MRI-visible CSVD markers.

*PECO* Population, Exposure, Comparator, Outcome, *CSVD* Cerebral Small Vessel Disease, *WMH* White Matter Hyperintensities, *MRI* Magnetic Resonance Imaging, *T1* T1-weighted Imaging, *T2* T2-weighted Imaging, *FLAIR* Fluid-Attenuated Inversion Recovery, *DTI* Diffusion Tensor Imaging, *DKI* Diffusion Kurtosis Imaging, *IVIM* Intravoxel Incoherent Motion Imaging, *ASL* Arterial Spin Labeling, *DWI* Diffusion-Weighted Imaging

### Data extraction and risk of bias assessment

The retrieved studies were checked for duplicates, and screening was performed using Rayyan (Ouzzani et al., 2016). The data extracted included author and publication year, study design, sample size, inclusion criteria, MRI technique, WMH assessment method, cognitive assessment tools used, statistical tests and main study results, including reported effect estimates, confidence intervals, and p-values wherever available. The study quality was checked using 11-item checklist recommended by Agency for Healthcare Research and Quality (AHRQ), with a total score of 11 (Ma et al., 2020). Studies with scores of 0–3, 4–7, and 8–11 were of low, moderate, and high quality, respectively. The quality assessment included domains such as the source of information, inclusion and exclusion criteria, patient recruitment, blinding, quality assurance, handling of exclusions and missing data, confounding control, follow-up completeness, and appropriateness of statistical analysis. Two reviewers independently conducted the quality assessment and resolved any disagreements through discussion.

### Data synthesis

The findings were synthesized narratively and were grouped according to the MRI techniques used, with conventional MRI and advanced MRI techniques considered separately. Within each group, findings were compared according to the cognitive domains assessed and the reported associations between MRI markers and the cognitive function. Studies reporting both adjusted and unadjusted findings were interpreted primarily using the adjusted results, while unadjusted findings were considered when adjusted estimate values were unavailable. Differences in the study populations, MRI techniques, cognitive assessment tools and statistical methods were considered when interpreting conflicting findings. The methodological quality of the included studies was also considered while interpreting the overall findings. No quantitative pooling or meta-analysis was performed.

## Results

### Study selection and study characteristics

**Fig. 1:**
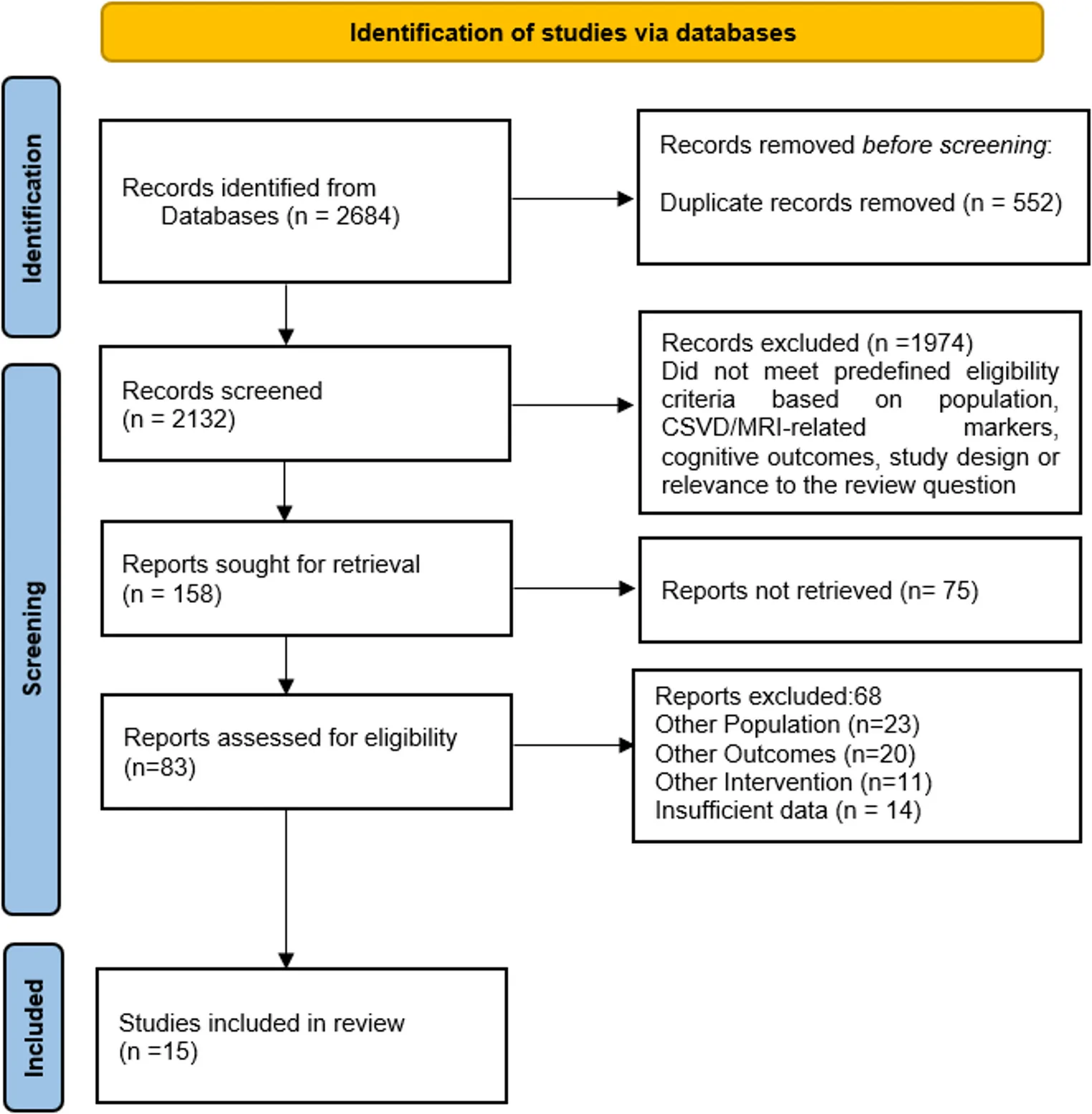
PRISMA flow diagram of study selection

A total of 2,684 articles were identified between 2016 and 2026, and 83 original articles remained after removing duplicates and screening titles and abstract. Among these only 15(Croall et al., 2017; Dobrynina et al., 2024; Feng et al., 2025; Hamilton et al., 2021; Lampe et al., 2019; Lin et al., 2023; Liu et al., 2023; Meng et al., 2026; Petersen et al., 2025; Vintimilla et al., 2021; Zeng et al., 2020; C. E. Zhang et al., 2017; Zhang et al., 2023; Zheng et al., 2025; Zhong et al., 2017) articles met the inclusion criteria and have been included in the review. The study selection process is shown in Fig. 1. This review consisted of prospective, cross-sectional and retrospective studies conducted across multiple centres with sample size between 54 and 2,603. Most of the studies used advanced MRI techniques in combination with conventional MRI techniques while very few employed conventional MRI sequences. Cognitive function was evaluated using the Mini-Mental State Examination (MMSE), the Montreal Cognitive Assessment (MoCA), and other validated cognitive assessment instruments. WMH severity assessment was done based on the volume, regional distribution periventricular white matter hyperintensities (PVWMH) and deep white matter hyperintensities (DWMH), tract involvement, Fazekas grading and quantitative diffusion measures. The details of study characteristics recorded from the included studies are given in Table 2.

**Table 2:** Characteristics of the included Studies

Author (Year)	Study Design	Sample Size	Age	Scanner/Field Strength	MRI Technique	Spatial Resolution	WMH Assessment	Cognition Tool/Domain	Effect Estimate (95% CI)	Outcomes Reported	Multiple-comparison Correction
Hamilton et al. (2021)	CS	540	72.6 ± 0.7	GE: 1.5T	T1W, T2W, T2*W, FLAIR	NR	WMH + total SVD burden	Multiple assessment tools	β = −0.371 (95% CI − 0.502 to − 0.240); β = −0.363 (95% CI − 0.493 to − 0.233)	Greater SVD burden associated with poorer cognition	FDR
Liu et al. (2023)	RP	104	MCI: 65; non-MCI: 64	Siemens: 3T	T1W, T2W, FLAIR, DWI, SWI, DKI, MRA	T1W: 0.9-mm isotropic; DWI/DKI: 3 × 3 × 3 mm³	WMH severity; CSVD; cingulum microstructure	MMSE, MoCA	DKI parameters associated with MoCA; 95% CI	Reduced DKI-derived parameters associated with poorer cognition	Bonferroni/Šídák-Bonferroni
Vintimilla et al. (2021)	CS	515	62.6 ± 7.7	Siemens: 3T	T1W/MPRAGE, FLAIR	NR	Quantitative WMH volume	Multiple assessment tools	MMSE: β = −0.43 (95% CI − 0.76 to − 0.10); DSST: β = −0.12 (95% CI − 0.22 to − 0.02)	Higher WMH volume associated with poorer executive function and cognition	NR
Croall et al. (2017)	PR	109	Mean 68	Multiple 3 T scanners	T1W, T2*W, FLAIR, DTI	DTI: 2-mm isotropic; T1: 1-mm isotropic	WMH + FA/MD	Multiple assessment tools	FA/MD associated with cognitive performance; 95% CI	Reduced FA and altered diffusivity associated with poorer cognition	NR
Dobrynina et al. (2024)	CS	210	CSVD: 60.4 ± 7.6; controls: 59.6 ± 6.8	Siemens: 3T	DTI, DKI	Diffusion: 2 × 2 × 2 mm³	WMH/CSVD; corpus callosum microstructure	Multiple assessment tools	AWF AUC = 0.89 (95% CI 0.84–0.94); RK AUC = 0.87 (95% CI 0.81–0.93)	Corpus callosum damage associated with cognitive dysfunction	NR
Zhong et al. (2017)	PR	75	67.05 ± 9.62	3T	DTI, DKI, ASL	NR	WMH + NAWM microstructure; CBF	MMSE	NAWM-FA: β = 0.403, *p* < 0.001; 95% CI NR	White matter integrity showed stronger associations with cognition than perfusion measures	NR
Zhang et al. (2023)	CS	99	70	Siemens: 3T	T1W, T2W, FLAIR, MRA	T1: 1.6 mm; T2/FLAIR: 5 mm	WMH volume; WMH/total-WM ratio	MoCA	WMH volume: OR = 1.195 (95% CI 1.074–1.330); WMH/WM ratio: AUC = 0.800 (95% CI 0.710–0.891)	Increased WMH volume independently predicted cognitive dysfunction	Benjamini–Hochberg/FDR
Lin et al. (2023)	PR	80	WMH: 70 ± 7.7; controls: 68 ± 6.2	Siemens: 3T	T1W, T2W, FLAIR, IVIM	T1W/T2W/FLAIR: 0.7 × 0.7 × 5 mm³; IVIM: 5-mm slice	DWMH, PWMH, NAWM; IVIM D, D*, f	MMSE, MoCA	D associated with WMH severity and cognitive scores; 95% CI	Increased diffusivity in severe WMH regions associated with poorer cognition	NR
Zheng et al. (2025)	RP	54	CI: 65.7 ± 9.4; non-CI: 71.8 ± 7.5	Philips: 3T	T1W, DTI	DTI: 2.5 × 2.5 × 2.5 mm³	WMH tracts; FA/MD/AD/RD	MMSE, MoCA	AUC = 0.940 training; 0.809 testing; 95% CI NR	DTI-based multivariate models predicted cognitive impairment and white matter injury	TFCE/FWE
Petersen et al. (2025)	PR	2,603	Median 65	Siemens: 3T	T1W, 3D FLAIR, DWI	FLAIR: 0.75 × 0.75 × 0.9 mm³; 0.9-mm slice	WMH load, Fazekas, PVS, PSMD	Multiple assessment tools	MD: β = 0.140 (95% CI 0.09–0.19); tissue FA: β = −0.097 (95% CI − 0.13 to − 0.06)	Higher CSVD burden associated with gray matter abnormalities and poorer cognition	FWE
Meng et al. (2026)	PR	128	65.1 ± 6.3	GE: 3T	T1W, FLAIR, 3D-pCASL/ASL	FLAIR: 6-mm slice	Fazekas WMH + regional CBF	MMSE, MoCA	CBF associated with WMH severity/cognition; 95% CI NR	Increased regional cerebral perfusion with increasing WMH severity, associated with lower cognitive performance.	NR
Zeng et al. (2020)	CS	321	66.4	NR	T2W, FLAIR, DTI	FLAIR: 3-mm slice	Fazekas WMH + quantitative WMH volume/LST	Multiple assessment tools	WMH severity associated with poorer memory; 95% CI NR	Severe WMH associated with memory deficits and white matter microstructural abnormalities	FWE/FDR
Zhang et al. (2017)	PR	112	SVD: 70 ± 11; controls: 69 ± 12	NR	T1W, T2W, FLAIR, IVIM	NR	WMH regions; IVIM perfusion	Multiple assessment tools	NR	Lower microvascular perfusion associated with poorer cognition in SVD	NR
Feng et al. (2025)	CC	100	CSVD: 69.1 ± 4.8; controls: 68.5 ± 5.2	GE: 3T	T1W, T2W, FLAIR, DWI, SWI, DTI, MRA	NR	Fazekas WMH + FA; CMBs	Multiple assessment tools	WMH severity/reduced FA associated with deficits; 95% CI	Greater WMH severity and reduced FA associated with executive and attention deficits	NR
Lampe et al. (2019)	CS	702	68.49 ± 4.58	Siemens: 3T	T1W/MPRAGE, FLAIR	1-mm isotropic	Automated WMH segmentation using Lesion-TOADS	Multiple assessment tools	Region-specific WMH associated with domain-specific deficits; 95% CI	Region-specific WMH distribution associated with domain-specific cognitive deficits	NR

*CS* cross-sectional, *PR* prospective, *RP* retrospective, *CC* case-control, *WMH* white matter hyperintensities, *SVD* small vessel disease, *CSVD* cerebral small vessel disease, *MRI* magnetic resonance imaging, *T1W/T1WI* T1-weighted imaging, *T2W/T2WI* T2-weighted imaging, *FLAIR* fluid-attenuated inversion recovery, *DWI* diffusion-weighted imaging, *SWI* susceptibility-weighted imaging, *DTI* diffusion tensor imaging, *DKI* diffusion kurtosis imaging, *MRA* magnetic resonance angiography, *ASL* arterial spin labeling, *IVIM* intravoxel incoherent motion, *D* true diffusion coefficient, D* pseudo-diffusion coefficient, *f* perfusion fraction, *FA* fractional anisotropy, *MD* mean diffusivity, *AD* axial diffusivity, *RD* radial diffusivity, *AWF* axonal water fraction, *RK* radial kurtosis, *CBF* cerebral blood flow, *NAWM* normal-appearing white matter, *PVS*, perivascular spaces, *PSMD* peak width of skeletonized mean diffusivity, *LST* Lesion Segmentation Toolbox, *MMSE* Mini-Mental State Examination, *MoCA* Montreal Cognitive Assessment, *DSST* Digit Symbol Substitution Test, *AUC *area under curve, *CI* confidence interval, *OR* odds ratio, *FDR* false discovery rate, *TFCE* threshold-free cluster enhancement, *FWE* family-wise error, *NR* not reported. Spatial resolution is reported according to the information provided in the original studies. Where complete voxel dimensions were available or could be derived from the reported field of view, acquisition matrix, and slice thickness, these are presented; otherwise, the reported slice thickness is provided

### Quality assessment of the study

**Fig. 2:**
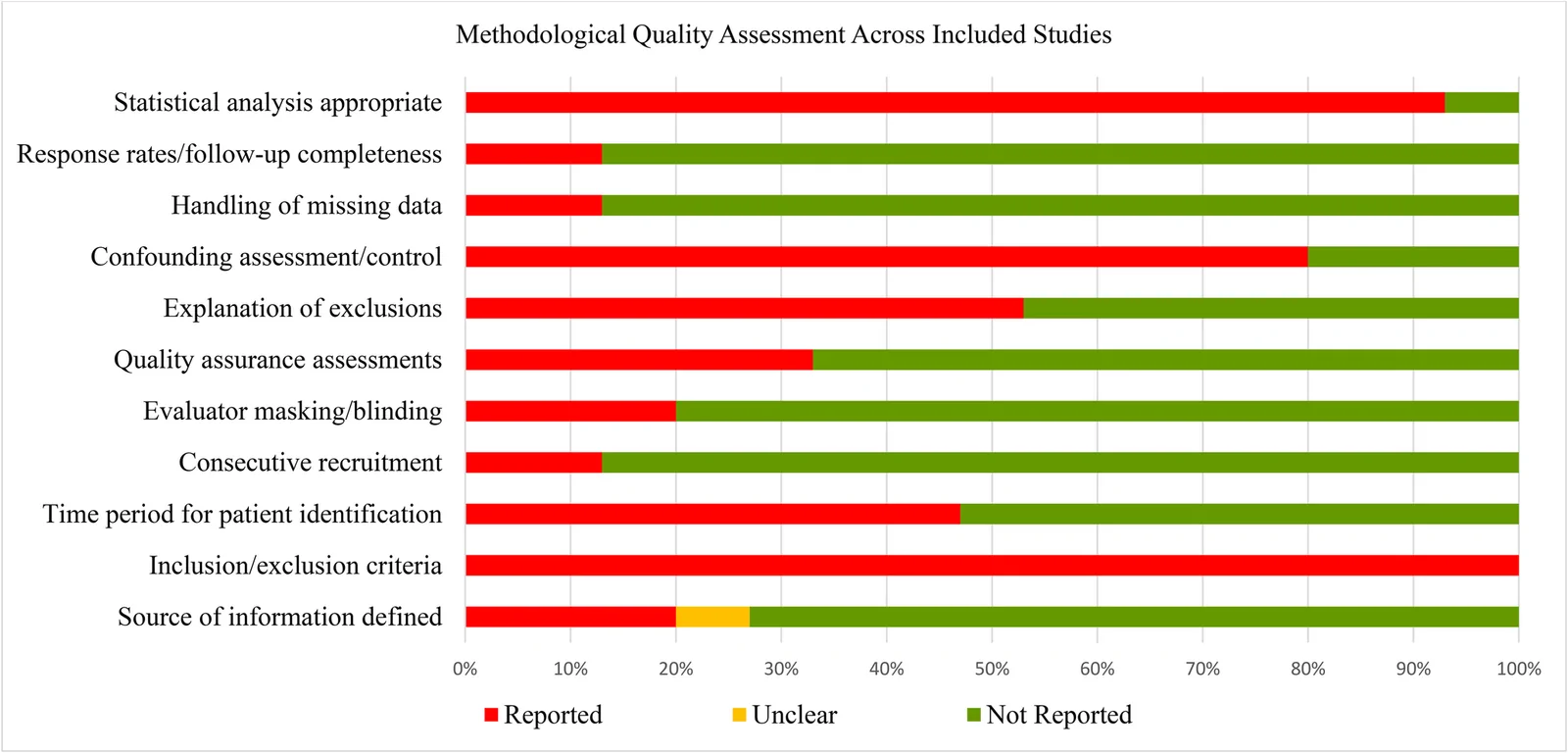
Methodological quality assessment of included studies

Three studies (Croall et al., 2017; Hamilton et al., 2021; Petersen et al., 2025) were considered high quality, with remaining 12 studies (Dobrynina et al., 2024; Feng et al., 2025; Lampe et al., 2019; Lin et al., 2023; Liu et al., 2023; Meng et al., 2026; Vintimilla et al., 2021; Zeng et al., 2020; C. E. Zhang et al., 2017; Zhang et al., 2023; Zheng et al., 2025; Zhong et al., 2017) were of moderate quality. The source of information, inclusion and exclusion criteria, and statistical analysis were clearly defined in all studies. In addition to this all studies addressed quality assurance and confounding assessments. However, limited or unclear reporting was observed for the time period of patient identification, handling of missing data, response rates/follow-up completeness, and explanations for exclusions. Information regarding consecutive recruitment and evaluator masking/blinding was also unclear across the included studies. A detailed summary of the methodological quality assessment is presented in Fig. 2. A supplementary table has been provided with individual quality assessment of the included studies.

### Conventional MRI markers of WMH and cognition in CSVD

In the studies reviewed, conventional MRI techniques demonstrated a consistent association between WMH burden and poorer cognitive performance. Cognitive declines were observed across various domains, with the most affected areas being processing speed, executive function, memory, visuospatial ability, motor speed, and general cognition. Poorer cognitive performance was associated with higher total SVD burden across all assessed domains (Hamilton et al., 2021). The strongest associations were observed for processing speed (β = −0.371, *p* < 0.001) and general cognition (β = −0.363, *p* < 0.001), followed by memory and visuospatial performance. The negative β values indicate that greater SVD burden was associated with poorer cognitive function. Similar findings were also reported by (Lampe et al., 2019), with a significant association across all domains. Additionally, the study reported that WMH in frontal regions were more closely associated with executive dysfunction, while parieto-temporal WMH was associated with poorer memory performance, and deep white matter lesions were associated with impaired motor speed. An increase in WMH volume was linked to poorer cognitive outcomes, including lower scores on the MMSE, Trails B, and DSST, with these associations observed even in cognitively normal individuals (Vintimilla et al., 2021). Increased WMH volume and higher WMH-to-white matter ratios associated with poorer visuospatial and memory performance, with ROC analysis showing good predictive performance area under curve (AUC = 0.800, 95% confidence interval (CI): 0.710–0.891) (Zhang et al., 2023).

### Advanced MRI markers of WMH and cognition in CSVD

Throughout the included studies, advanced MRI techniques like diffusion tensor imaging (DTI), diffusion kurtosis imaging (DKI), intravoxel incoherent motion (IVIM), and arterial spin labeling (ASL) showed correlations between altered cerebral perfusion, white matter microstructural abnormalities, and cognitive impairment. Cognitive impairments were commonly observed across domains such as executive function, memory, attention, processing speed, and global cognition. Studies using DTI reported consistent associations between disrupted white matter integrity, characterized by reduced fractional anisotropy (FA) and increased diffusivity measures, and poorer cognitive performance (Croall et al., 2017; Zeng et al., 2020). Higher white matter FA was positively associated with cognitive performance, including global cognition (β = 0.268), mental flexibility (0.306), verbal fluency (β = 0.376) and MoCA performance (β = 0.273) (Croall et al., 2017). The positive β values indicate that higher FA was associated with better cognitive performance, whereas lower FA was associated with poorer cognitive performance. Similar findings were reported in the study by (Zeng et al., 2020), which found that increasing WMH severity was associated with reduced memory with decreased FA across multiple white matter tracts. Study also showed that patients with mild WMH showed limited cognitive decline. Additionally, greater WMH volume was associated with reduced FA in NAWM (*r*=−0.443, *p* < 0.001) and increased MD in NAWM (*r* = 0.293, *p* = 0.012), indicating microstructural abnormalities beyond visible WMH (Zhong et al., 2017). Global cognitive function was independently associated with NAWM FA (β = 0.403, *p* < 0.001) and WMH FA (β = 0.211, *p* = 0.017). A model including NAWM-FA, WMH-FA and years of education explained 49% of variance in MMSE scores.

Likewise, (Zheng et al., 2025) identified reduced FA in several white matter tracts among CSVD patients with cognitive impairment, along with increased MD and RD in multiple regions. FA was analysed as a microstructural imaging marker, and significant FA measures were included as predictors, together with MD, RD and clinical variables in a multivariate logistic regression model. The model demonstrated good predictive performance, with mean AUCs of 0.940 in the training set and 0.809 in the testing set. The study by (Petersen et al., 2025) found that higher CSVD burden was associated with increased MD (β = 0.140) and free water (β = 0.183) as well as reduced tissue FA (β=−0.097), T1/FLAIR ratio (β= −0.090) and cortical thickness and volume (β=−0.051). These imaging alterations were associated with lower general cognitive ability. (Feng et al., 2025) used multiple MRI techniques, including DTI, and found that WMH affected executive function and attention, with lower MoCA, Digit Span Test (DST), and Color Trail Test (CTT-A/B) scores compared with controls (all *p* < 0.05). Studies using diffusion kurtosis imaging (DKI) demonstrated that reduced diffusion-derived parameters, including FA, axial kurtosis, mean kurtosis, radial kurtosis, and kurtosis fractional anisotropy, were associated with lower MoCA scores and poorer cognitive performance. The study by (Liu et al., 2023) reported lower MoCA scores with a significant group difference (*p* = 0.038) and moderate correlations with cognitive performance (*r* = 0.310–0.441). Study also reported abnormalities of the anterior cingulum strongly associating with attention and executive dysfunction, whereas learning and episodic memory were linked with parahippocampal cingulum abnormalities. Similarly, the study by (Dobrynina et al., 2024) reported progressive corpus callosum microstructural alterations across increasing cognitive impairment severity, from subjective cognitive impairment to mild cognitive impairment and dementia. Among the diffusion derived measures, axonal water fraction (AWF) showed the highest diagnostic performance (AUC = 0.89, 95% CI: 0.84–0.94), followed by RK (AUC = 0.87, 95% CI: 0.81–0.93) and FA (AUC = 0.86, 95% CI: 0.81–0.92).

Across IVIM studies, microstructural damage and vascular disruptions were associated with cognitive decline, with altered diffusion and perfusion parameters, particularly increased parenchymal diffusivity (D) and reduced microvascular perfusion (D*) and perfusion fraction (f). (Lin et al., 2023) reported that increased D and reduced D* and f within WMH regions were associated with poorer cognitive performance, with significantly lower MMSE and MoCA scores in patients compared with controls with p value < 0.001 and < 0.05, respectively. The study also demonstrated increased D and lower D* values in the NAWM region, suggesting that microstructural and microvascular abnormalities may extend beyond visibly affected WMH regions. However, (C. E. Zhang et al., 2017) reported reduced D* and f in NAWM and cortical gray matter rather than D that associated with poorer overall cognition, executive function, and information-processing speed (*p* = 0.03, *p* = 0.002, and *p* = 0.04, respectively), suggesting vascular dysfunction as a key contributor to cognitive impairment in cerebral small vessel disease. Lastly, studies using ASL (Meng et al., 2026) reported regional differences in cerebral blood flow (CBF) across increasing Fazekas grades, with higher CBF in the bilateral lenticular nuclei and left thalamus/hippocampus among participants with greater WMH severity. Higher CBF in these regions was negatively correlated with cognitive performance, including MMSE and MoCA scores, with correlation coefficients ranging from − 0.177 to −0.306 for MMSE and − 0.1179 to −0.269 for MoCA (*p* < 0.047).

## Discussion

This review investigated the association between MRI markers of WMH in CSVD and cognitive performance. Our study shows that worse cognitive performance is associated with a higher burden of WMH and CSVD across domains, including executive function, processing speed, memory, visuospatial ability, attention, and global cognition. Both conventional and advanced neuroimaging markers showed a consistent relationship with cognitive impairment along with microstructural damage, altered diffusion and perfusion patterns, and disrupted white matter integrity in cognitive decline.

WMH are hallmark features of cerebral small vessel disease and are frequently observed in older individuals, with a greater WMH burden associated with cognitive decline and dementia (Debette & Markus, 2010; Østergaard et al., 2016; Prins & Scheltens, 2015). Conventional structural MRI, which identifies WMH as hyperintense lesions on T2/FLAIR sequences, provides a macroscopic view of disease burden. WMH are heterogeneous lesions occurring due to different brain parenchymal changes, and their burden increases progressively with age (Fazekas et al., 1991, 1993; Gouw et al., 2011). Our findings suggest that greater WMH burden is associated with poorer cognitive performance, particularly in global cognition, executive function and processing speed among individuals with CSVD. The association was observed across multiple cognitive domains, indicating that WMH-related damage is not limited to isolated functions but reflects broader disruption of brain processes. These findings are consistent with the previous study (Roseborough et al., 2023) which reported that WMH burden is associated with an increased risk of cognitive impairment and dementia across different populations. While previous studies have often emphasized total WMH volume, our findings suggest that cognitive impairment is not solely dependent on lesion burden but may also be influenced by regional distribution. This observation aligns with the previous study (Markus & Erik de Leeuw, 2023), which examined the spatial heterogeneity of WMH and its differential impact on cognition. Conventional structural MRI identifies WMH as hyperintense lesions on T2/FLAIR sequences but cannot detect microstructural damage that occurs before lesions become visible.

Advanced imaging techniques, particularly diffusion tensor imaging (DTI), have been utilized to detect the integrity of white matter tracts beyond what is visible on structural MRI. Our findings are consistent with those reported by (Xie et al., 2022), which reported that regional white matter alterations detected by DTI were associated with domain specific cognitive deficits, with lower FA and higher MD, indicating greater cognitive impairment. Similarly, reduced structural network efficiency derived from diffusion imaging was independently associated with cognitive impairment, even after accounting for conventional MRI markers (Tuladhar et al., 2016). This suggests that WMH may impair cognition not only through focal lesions but by disrupting the efficiency of large-scale brain networks. A network-based framework was proposed in which structural alterations associated with CSVD affected multiple cognitive domains through distributed connectivity changes (Zhang et al., 2026). These findings suggest that cognitive impairment in CSVD is associated with microstructural damage and disrupted communication between different brain regions. Additionally, diffusion imaging can also detect abnormalities in normal-appearing white matter, indicating that pathological changes extend beyond visible WMH, which may explain why structural lesion burden alone often underestimates cognitive impairment. Perfusion-based imaging techniques such as arterial spin labeling (ASL) provide complementary information by assessing cerebral blood flow and vascular function, although direct ASL- based evidence are limited within the included studies. Our findings are consistent with those reported by (Statema et al., 2025), which showed that reduced cerebral blood flow in WMH regions was associated with cognitive impairment. The study using ASL and DTI suggested that combining microstructural and perfusion imaging shows microstructural disruption and hypoperfusion that are highly associated with cognitive impairment in the regions of WMH and in NAWM (Han et al., 2024). In contrast the included evidence showed that increased regional CBF with increasing WMH severity was associated with poorer cognitive performance. Similarly, CSVD has been described as a disorder of the neurovascular unit, where impaired vascular regulation associated with chronic hypoperfusion and subsequent white matter damage (Cannistraro et al., 2019). The findings suggest that perfusion abnormalities are associated with structural changes and cognitive impairment in CSVD. Our findings from advanced, non-contrast MRI techniques indicated that IVIM- and DKI- derived perfusion and diffusion parameters were associated with microvascular and microstructural abnormalities and cognitive performance. IVIM parameters, including an increased true diffusion coefficient (D) and decreased perfusion fraction were associated with cognitive decline, findings that are consistent with relationships reported between diffusion parameters and WMH burden (Wong et al., 2020). Similarly, cognitive impairment in CSVD results from combined vascular dysfunction and widespread brain network disruption rather than isolated WMH lesions (Paschoal et al., 2018). Another non-invasive diffusion-based technique DKI, is used to assess microstructural tissue complexity and heterogeneity using parameters such as mean kurtosis (MK) and mean diffusivity (MD), which have also shown strong correlations with cognitive impairment with increased MD and decreased MK. Thalamic microstructural alterations were evaluated, and changes in non-Gaussian diffusion were associated with cognitive decline among CSVD patients (Li et al., 2025). Additionally, DKI parameters can detect abnormalities even in normal-appearing white matter (Guo et al., 2025). Our findings show that even though multiple MRI techniques have been used to study WMH association with cognition multimodal studies are essential for explaining cognitive impairment in CSVD.

This systematic review has several strengths. First, this review provides a focused synthesis of evidence from both conventional and advanced MRI techniques, including structural MRI, DTI, ASL, IVIM, and DKI, to evaluate the association between WMH and cognitive impairment in CSVD. Unlike previous studies that focused on a single imaging modality, this review focuses on the association between structural, microstructural, and vascular abnormalities and cognitive performance. Second, strict inclusion criteria were applied, and only studies with clearly defined imaging markers and standardized cognitive assessments were included, which improves the reliability of the findings. Third, the methodological quality of the included studies was systematically assessed using the AHRQ checklist, ensuring transparency in evaluating risk of bias. In addition, organizing the findings based on imaging modality allowed for a clearer interpretation of how different MRI techniques contribute to understanding WMH-related cognitive impairment. Importantly, this review goes beyond simple description by organizing findings based on imaging modality, allowing for a clearer comparison of how different MRI techniques relate to cognition. The review identifies consistent associations between diffusion- based imaging measures and cognitive performance across several studies, although differences in imaging protocols and statistical approaches limited direct comparison between MRI techniques. Another strength is the inclusion of the advanced MRI techniques, which were not frequently synthesized in previous reviews, thereby adding novelty and advancing current understanding in this field. Furthermore, by focusing specifically on WMH and their relationship with cognition in CSVD, rather than broader or mixed neurological conditions, this review provided clinically relevant understandings of imaging markers associated with cognitive decline. The integration of multimodal imaging evidence and emphasis on both microstructural and vascular mechanisms makes this study particularly impactful for future research and clinical applications.

Despite several strengths, this study also has several limitations. Firstly, there was considerable heterogeneity across studies in terms of imaging protocols, MRI parameters, and cognitive assessment tools used, which prevented quantitative meta-analysis and may have influenced the consistency of findings. Secondly, variability in reporting of imaging markers, including differences in WMH quantification methods and regional analysis, may have introduced bias in interpreting results. Furthermore, important confounding factors such as age, vascular risk factors, and comorbidities were not consistently adjusted across all studies. Another limitation is that some advanced imaging techniques were represented by a smaller number of studies, which may have limited the generalizability of these findings. The lack of multicentre validation using multimodal MRI imaging markers emphasizes the need for future research with larger sample sizes to better understanding of progression of CSVD and its impact on cognition.

## Conclusion

This systematic review showed that greater WMH burden was associated with poorer cognitive performance across multiple cognitive domains in individuals with CSVD. Higher WMH burden was also associated with an increased risk of cognitive impairment, suggesting that WMH may provide an indication to future cognitive outcomes in addition to capturing existing brain injury. Early detection of CSVD would help to reduce or delay the progression to dementia and stroke. While structural measures primarily reflect overall lesion burden, advanced diffusion techniques appear to capture subtle microstructural disruption that relates more closely to cognitive performance across domains. Therefore, future research should focus on large, prospective, longitudinal studies with standardized imaging and analysis methods to establish the value of these imaging markers for early identification and monitoring of cognitive impairment in CSVD.

## Supplementary Information

Below is the link to the electronic supplementary material.

Supplementary Material 1

Supplementary Material 2

## Data Availability

The data will be available on request.
