# Supplementary material for: Multimodal MRI markers of white matter hyperintensities and their impact on cognition in cerebral small vessel disease: a systematic review
Source: Brain Imaging Behav. 2026 Oct 8;20(5):137. doi: 10.1007/s11682-026-01209-8 (PMC13649864; doi:10.1007/s11682-026-01209-8)
Supplement: Supplementary file 1 [file 11682_2026_1209_MOESM1_ESM.docx]

**Pubmed**

( ("Cerebral Small Vessel Disease"[Mesh] OR "cerebral small vessel disease" OR CSVD OR "small vessel disease" OR "white matter hyperintensities" OR WMH OR "white matter lesion*" OR "leukoaraiosis") ) AND ( "MRI"[Mesh] OR "magnetic resonance imaging" OR MRI OR T1 OR T2 OR FLAIR OR DTI OR "diffusion tensor imaging" OR DKI OR "diffusion kurtosis imaging" OR IVIM OR "intravoxel incoherent motion" OR ASL OR "arterial spin labeling" OR DWI OR "diffusion weighted imaging" ) AND ( "cognitive impairment" OR cognition OR "cognitive decline" OR "cognitive dysfunction" OR "executive dysfunction" OR memory ) NOT ( "brain tumor*" OR Alzheimer* OR "Parkinson disease" OR "multiple sclerosis" OR stroke )

**Web of Science**

((ALL="Cerebral Small Vessel Disease" OR "cerebral small vessel disease" OR CSVD OR "small vessel disease" OR "white matter hyperintensities" OR WMH OR "white matter lesion*" OR leukoaraiosis )) AND (ALL=MRI OR "magnetic resonance imaging" OR MRI OR T1 OR T2 OR FLAIR OR DTI OR "diffusion tensor imaging" OR DKI OR "diffusion kurtosis imaging" OR IVIM OR "intravoxel incoherent motion" OR ASL OR "arterial spin labeling" OR DWI OR "diffusion weighted imaging" ) AND ("cognitive impairment" OR cognition OR "cognitive decline" OR "cognitive dysfunction" OR "executive dysfunction" OR memory ) NOT ("brain tumor*" OR Alzheimer* OR "Parkinson disease" OR "multiple sclerosis" OR stroke )

**Scopus**:

(INDEXTERMS("Cerebral Small Vessel Disease") OR "cerebral small vessel disease" OR CSVD OR "small vessel disease" OR "white matter hyperintensities" OR WMH OR "white matter lesion*" OR leukoaraiosis )) AND (INDEXTERMS(MRI) OR "magnetic resonance imaging" OR MRI OR T1 OR T2 OR FLAIR OR DTI OR "diffusion tensor imaging" OR DKI OR "diffusion kurtosis imaging" OR IVIM OR "intravoxel incoherent motion" OR ASL OR "arterial spin labeling" OR DWI OR "diffusion weighted imaging" ) AND ("cognitive impairment" OR cognition OR "cognitive decline" OR "cognitive dysfunction" OR "executive dysfunction" OR memory ) NOT ("brain tumor*" OR Alzheimer* OR "Parkinson disease" OR "multiple sclerosis" OR stroke )

**Embase**

(('Cerebral Small Vessel Disease'/exp OR 'cerebral small vessel disease' OR CSVD OR 'small vessel disease' OR 'white matter hyperintensities' OR WMH OR 'white matter lesion*' OR leukoaraiosis )) AND (MRI/exp OR 'magnetic resonance imaging' OR MRI OR T1 OR T2 OR FLAIR OR DTI OR 'diffusion tensor imaging' OR DKI OR 'diffusion kurtosis imaging' OR IVIM OR 'intravoxel incoherent motion' OR ASL OR 'arterial spin labeling' OR DWI OR 'diffusion weighted imaging' ) AND ('cognitive impairment' OR cognition OR 'cognitive decline' OR 'cognitive dysfunction' OR 'executive dysfunction' OR memory ) NOT ('brain tumor*' OR Alzheimer* OR 'Parkinson disease' OR 'multiple sclerosis' OR stroke )
