# Supplementary material for: Multimodal MRI markers of white matter hyperintensities and their impact on cognition in cerebral small vessel disease: a systematic review
Source: Brain Imaging Behav. 2026 Oct 8;20(5):137. doi: 10.1007/s11682-026-01209-8 (PMC13649864; doi:10.1007/s11682-026-01209-8)
Supplement: Supplementary file 2 [file 11682_2026_1209_MOESM2_ESM.docx]

**Risk of Bias: AHRQ**

| **S. No.** | **Author** | **Source of information defined** | **Inclusion/exclusion criteria** | **Time period for patient identification** | **Consecutive recruitment** | **Evaluator masking/blinding** | **Quality assurance assessments** | **Explanation of exclusions** | **Confounding assessment/control** | **Handling of missing data** | **Response rates/follow-up completeness** | **Statistical analysis appropriate** | **Score** |
| --- | --- | --- | --- | --- | --- | --- | --- | --- | --- | --- | --- | --- | --- |
| 1 | Hamilton et al. (2021) | 1 | 1 | 0 | 0 | 0 | 1 | 1 | 1 | 1 |  | 1 | 8 |
| 2 | Liu et al. (2022) | 1 | 1 | 0 | 0 | 0 | 1 | 0 | 1 | 0 | 0 | 1 | 5 |
| 3 | Vintimilla et al. (2021) | 1 | 1 | 0 | 0 | 0 | 1 | 0 | 1 | 0 | 1 | 1 | 6 |
| 4 | Croall et al. (2017) | 1 | 1 | 0 | 0 | 0 | 1 | 1 | 1 | 1 |  | 1 | 8 |
| 5 | Dobrynina et al. (2024) | 1 | 1 | 0 | 0 | 0 | 1 | 0 | 1 | 0 |  | 1 | 6 |
| 6 | Zhong et al. (2017) | 1 | 1 | 0 | 0 | 0 | 1 | 0 | 1 | 0 | 0 | 1 | 5 |
| 7 | Zhang et al. (2023) | 1 | 1 | 0 | 0 | 0 | 1 | 0 | 1 | 0 | 0 | 1 | 5 |
| 8 | Lin et al. (2023) | 1 | 1 | 0 | 0 | 0 | 1 | 0 | 1 | 0 | 0 | 1 | 5 |
| 9 | Zheng et al. (2025) | 1 | 1 | 0 | 0 | 0 | 1 | 0 | 1 | 0 | 0 | 1 | 5 |
| 10 | Petersen et al. (2025) | 1 | 1 | 1 | 0 | 0 | 1 | 1 | 1 | 1 | 1 | 1 | 9 |
| 11 | Meng et al. (2026) | 1 | 1 | 0 | 0 | 0 | 1 | 0 | 1 | 0 | 0 | 1 | 5 |
| 12 | Zeng et al. (2020) | 1 | 1 | 0 | 0 | 0 | 1 | 0 | 1 | 0 | 0 | 1 | 5 |
| 13 | Zhang et al. (2017) | 1 | 1 | 0 | 0 | 0 | 1 | 0 | 1 | 0 | 0 | 1 | 5 |
| 14 | Feng et al., 2025 | 1 | 1 | 0 | 0 | 0 | 1 | 1 | 1 | 0 | 0 | 1 | 6 |
| 15 | Lampe et al., 2019 | 1 | 1 | 1 | 0 | 0 | 1 | 0 | 1 | 0 | 0 | 1 | 7 |
